# Sustainability, cooperation and mobility of workers within and between European countries: a two-stage goal programming model

**DOI:** 10.1007/s10479-020-03818-y

**Published:** 2020-10-12

**Authors:** Danilo Liuzzi, Veronica Lupi, Aymeric Vié

**Affiliations:** 1 Department of Economic, Business, Mathematical and Statistical Sciences, University of Triste, Trieste, Italy; 2 GREEN, Centre for Geography, Resources, Environment, Energy and Networks, Bocconi University, Milan, Italy; 3 Paris School of Economics, Paris, France; 4 New England Complex Systems Institute, Cambridge, MA USA; 5 Sciences Po Saint-Germain-en-Laye, Saint-Germain-en-Laye, France

**Keywords:** Cooperation, Europe 2020 Agenda, Multiple criteria decision analysis, Goal programming, Migration, Sustainable development, C61, C63, E61, F22

## Abstract

Facing multiple and often considered as conflicting stakes, either economical, migratory, or environmental, policy-making may struggle to identify and implement relevant policy action allowing for balanced and joint completion of such challenges. Addressing this important public issue, we develop a multi-criteria two-stage Goal Programming (GP) model to identify optimal policy paths towards the Europe 2020 strategy on economic growth, employment levels and environmental sustainability. The model is calibrated on current contributions of economic sectors in all European countries to each policy objective: contribution to economic output (GDP), emissions of Green House Gas, electric consumption and number of jobs. First, we study the optimal allocation of workers within economic sectors of each European country to maximize the joint achievement of Europe 2020 multi criteria sustainability targets. We then extend the model to allow cooperation between states, namely allowing internal migrations of workers between countries. We highlight how supranational allocation schemes of surplus workers improve the satisfaction of national sustainability objectives. Finally, we consider extra-European migrants regional integration and study the consequences of such opening over EU2020 targets satisfaction and per capita GDP. Simulation results highlight countries performance comparison, and sheds light on significant benefits from such cooperation for the majority of countries. Improved integration of internal and external workforce generally improves the achievement of EU2020 objectives, while keeping per capita GDP at least constant. Moreover, we expose the relevance of cooperative work-flows allocation strategies across Europe and emphasize the importance of workers mobility in order to ensure more sustainable common development.

## Introduction

Nowadays, policymakers are puzzled as to find a compromise between economic, environmental, energy and social targets. Public awareness on these topics has been raising in the last years and significant shares of public opinion require their governments to find the right balance between sustainable development and economic growth. In order to address this issue, in 2010 the European Commission established the Europe 2020 strategy (European Commission 2010). This is a policy package designed to recover from the 2007/2008 crisis. Generally speaking, it aims at overcoming the structural weaknesses of the European economy, improving its competitiveness and underpinning a sustainable market economy. The main objectives of the EU2020 strategy are collected in an agenda, which identifies several tasks to be completed throughout the coming decade. It covers a high variety of interrelated targets in several domains, including economy, public investment, education, reduction of Green House Gases (GHG) emissions, energy efficiency, employment, poverty and social inclusion. In particular, by 2020 the European Union should: (i) decrease GHG emissions by 20% with respect to 1990 levels; (ii) 20% of energy should come from renewable sources; (iii) energy efficiency has to increase by 20%; (iv) the employment rate of people between 20 and 60 years is expected to be 75%. These tasks should be transposed to national objectives, calling for policy design and recommendations for each European country that are able to solve the conflicts arising between these objectives.

Multiple Criteria Decision Analysis (MCDA) offers relevant tools to tackle such issues. One of its methods, the Goal Programming (GP) methodology, allows to identify the optimal allocation of decision variables with respect to one or several criteria, granting a minimal deviation from specified targets by linear programming. GP has been widely used across several domains, as (Colapinto et al. 2015) outlined in a comprehensive review study. Notably, GP models have been applied to engineering problems such as supply chain optimization (Selim and Ozkarahan 2008), vendor selection problem (Kumar et al. 2004; Zhou et al. 2000, 2006), production-distribution planning (Selim and Ozkarahan 2008), manufacturing and production decision making (Sheikhalishahi and Torabi 2014; Taghizadeh et al. 2011). The GP approach and variants have also been used in financial portfolio selection and management (Watada 1997; Inuiguchi and Ramik 2000), or also media planning for marketing (Jha et al. 2011).

Goal programming has also been applied to macroeconomic policy design and evaluation. Previous important work using MCDA methods and more specifically goal programming can be traced back to Wallenius (1982). There exists a recent and growing trend in the Goal-Programming literature aiming to apply these methodologies to policy-making and policy design. Some researchers (Jayaraman et al. 2015b, 2017b, 2015a, 2017a) use different Goal-Programming and derived approaches to study global sustainability and development of the Persian Gulf countries. These analyses rely on the identification of individual worker contribution to Gross Domestic Product (GDP), electric consumption, GHG emissions per economic sector, and allocation of these workers in the different economic sectors to satisfy designated targets in each criteria. Individual contribution stands for the ratio of a given economic sector share of GDP, electric consumption, or GHGs emissions, over the number of workers employed by the sector considered.

While it is intuitive that this engineering workers’ allocation approach encounters limits, due to the imperfect mobility of human capital due to preferences, qualifications and individual choices, it should be outlined that the development of economic sectors is a major means of action for policy makers, through investments in sectors, education, and in professional mobility. This is why this literature often assumes, starting from the year of analysis, that the number of workers in an economic sector is constant in the final period with respect to the year of analysis. This social goal aims at not destroying current jobs, only allocating the incoming population from natural population growth, workers mobility, and migration.

Other authors employ these techniques to study macroeconomic policies in Spain (San Cristóbal 2012). More recently, a study about global sustainability of all European Union countries using Fuzzy Goal Programming methodologies has been proposed in Vié et al. (2018). Nomani et al. (2017) implemented a Fuzzy Goal Programming approach to evaluate the satisfaction of sustainability policy targets in India. Omrani, Valipour and Emrouznejad created a weighted Goal Programming model to plan efficiently regional sustainable development and workforce allocation in Iran, see Omrani et al. (2018). These Goal Programming and associated models have often been implemented for specific countries or regions with few countries in the context of comparisons as in Jayaraman et al. (2015a). Some papers have instead focused on evaluating the Europe 2020 objectives framework over a larger number of countries (28). Our analysis builds on the work of Vié et al. (2018) and Liuzzi et al. (2020) on sustainability of European Union countries under the Europe 2020 paradigm.

In this work we implement a two-stage optimization process to analyze EU2020 targets sustainability, in the continuity of the recent trends in employing GP analysis to optimal policy design facing various constraints. In particular, in continuity with the previous literature, we focus on the optimal allocation of workers in the European countries. In our optimization procedure, we notice that most of the EU countries would benefit from a larger workforce, while few states require a shrink in the number of available workers. Hence, we relax our constraints to allow for migration within the European Union. To the best of our knowledge, no related research has yet included migrations and supranational cooperation in the analysis of macroeconomic policies using MCDA methods. We aim to shed lights on the importance of free movement intra and extra Europe, highlighting the trade-off regarding environmental policy, social welfare and economic growth. Moreover, in the optimal allocations for satisfaction of Europe 2020 objectives identified by Liuzzi et al. (2020) and Vié et al. (2018), we highlight that the excess worker supply provided within EU countries is not always enough to satisfy the workforce requirements. Facing the current increase in migration flows towards Europe due to economic, political and international contexts, the transition towards sustainable development and reduction of environmental impact cannot ignore these migration flows. Thus, we investigate the consequences of allowing extra-Europe immigration in the model over the satisfaction of Europe 2020 policy targets. The main contribution of this work is to include migration flows in the design of multi-criteria policies.

In particular, the first linear programming stage determines the optimal national allocations for each country using a standard macroeconomic GP model à la Jayaraman et al. (2017b) with several criteria and economic sectors. Two “national policy plans” are determined, one including the possibility of workers flow (‘open’ plan) and one restricted to national workers (‘closed’ plan). A country adopts an open plan, namely a reallocation of workers, only if the objective function decreases and per capita GDP increase, i.e., if cooperating makes the country better off in both Europe 2020 objectives attainment and per capita wealth. In the second stage we introduce cooperation between countries, inasmuch as some countries are better off due to the adoption of an open plan. Under cooperation, countries can receive additional workforce from the ones in surplus, illustrating European workers working in other countries to tackle unemployment in their home country. This new allocation proceeds by maximizing a necessity fulfillment criterion balanced with the constraint of maintaining per capita wealth at least equal to the one reached in the closed plan. In other words, workers unemployed in their countries are offered in the model the possibility of filling a vacancy in a different country, provided that this marginal worker contribution does not reduce per capita GDP in the different country with respect to a migration-free scenario.

The paper is organized as follows. Section 2 describes our two stages model formulation including presentation of criteria, economic sectors, data collection procedures and analysis steps. Section 3 presents the results and Sect. 4 their implications. Section 5 concludes.

## Model formulation

### The standard Goal Programming methodology

The Goal Programming (GP) model was first presented by Charnes and Cooper (1952, (1955) and later developed by them in Charnes and Cooper (1961). Given a vector of *p* criteria determined by the functions $[f_i(X), f_2(X), ..., f_p(X)]$, and a vector of objectives $[g_1, g_2, ..., g_p]$, GP models seek to minimize the deviations between objectives and criteria, under the condition that *X*, the decision variable, lies in *D*, where *D* is a feasibility set. The standard formulation of the GP model introduced in Charnes and Cooper (1955) yields:

1a $$\begin{aligned} \text {minimize}\quad&\sum _{i=1}^{p}{\delta _i^++\delta _i^-} \end{aligned}$$

1b $$\begin{aligned} \text {subject to}\quad&f_i(X) + \delta _i^- - \delta _i^+ = g_i \quad \text {for} \; i=1,\ldots , p, \end{aligned}$$

1c $$\begin{aligned}&X\in D, \end{aligned}$$

1d $$\begin{aligned}&\delta _i^{\pm } \ge 0, i=1,\ldots , p \end{aligned}$$

where $\delta _i^-$ and $\delta _i^+$ are the positive and negative deviations with respect to the targeted goal levels $g_i$ for $i = 1, \dots ,p$. *D* is the feasible set in which the decision variable *X* must belong. Here, the result of the GP model expressed in Eqs. ((1a)–(1c)) returns a Pareto optimal solution in which it is impossible to improve the achievement of one criterion without harming the achievement of another. Several variants of this standard optimization model were developed; see Jones and Tamiz (2010) for a more detailed review.

### Model framework, decision variables and criteria

We investigate the framework of Europe 2020 (EU2020) policy targets in a diversity of scenarios about cooperation, accounting for policy-maker satisfaction and national preference. From the standard GP formulation outlined in Eqs. (1a)–(1c), we now provide details about the criteria $$f_i(X)$$ for $$i=1\ldots 4$$, the goals $$ G_i$$ for $$i=1\ldots 4$$ and the feasible set $$X\in D$$. In line with the above formulation , we introduce a linear model with 7 economic sectors and 4 criteria, calibrated with Eurostat data, namely the official accounting organization of the European Union. We are interested in the Pareto optimal allocation of workers in the context of EU2020 goals, and studying the possible benefits from supranational cooperation. Our model does not distinguish between skilled and unskilled workers and assumes full employment, i.e., the labour force is equal to total population. Moreover, there is perfect mobility of workers across sectors.

In a given country, $X_j$ is the number of workers in the j-th economic sector and represents the associated decision variable. We denote the aggregation of information, communication, financial and insurance activities as commercial services, and the administrative, state, technical, scientific, education, health and social services as general services. Table 1 shows the economic sectors considered in the model. The economic sectors and aggregates considered in the model are presented in Table 1. The selection of our 7 economic sectors is closely related to previous macroeconomic GP models (notably San Cristóbal 2012; Jayaraman et al. 2015a) and fits the economic activity classification system NACE Rev. 2 developed by the European Union.

**Table 1:** Economic sectors considered in the model

Decision variable	Sector
$X_1$	Agriculture, forestry and fishing
$X_2$	Energy industry
$X_3$	Manufacturing industry
$X_4$	Construction and residential
$X_5$	Trade, transports, distribution and repairing
$X_6$	Commercial services
$X_7$	General services

According to the EU2020 objectives, we formulate a macroeconomic GP model which simultaneously considers four criteria presented in Table 2 below.

**Table 2:** Europe 2020 main sustainability criteria

Criteria	Units
$F_1$	Economic output (in thousand EU €)
$F_2$	GHG emissions (in Gg of CO$_2$ equivalent kilo tons)
$F_3$	Electric consumption (in thousand tons of oil equivalent)
$F_4$	Number of employees (in thousands)

We assume that each criterion is linear with respect to the decision variable $X_j$:

2a $$\begin{aligned} F_1\left( X_1,X_2,\ldots ,\ X_7\right) =A_{11}X_1+A_{12}X_2+{\cdots +A}_{17}X_7 \end{aligned}$$

2b $$\begin{aligned} F_2\left( X_1,X_2,\ldots ,\ X_7\right) =A_{21}X_1+A_{22}X_2+{\cdots +A}_{27}X_7 \end{aligned}$$

2c $$\begin{aligned} F_3\left( X_1,X_2,\ldots ,\ X_7\right) =A_{31}X_1+A_{32}X_2+{\cdots +A}_{37}X_7 \end{aligned}$$

2d $$\begin{aligned} F_4\left( X_1,X_2,\ldots ,\ X_7\right) =A_{41}X_1+A_{42}X_2+{\cdots +A}_{47}X_7 \end{aligned}$$

This simplifying linearity assumption is consistent with the literature on economic and environmental policy design with MCDA (San Cristóbal 2012; Jayaraman et al. 2015b, 2017b, 2015a, 2017a; Vié et al. 2018). This follows the intuition that the total fulfilment of a given criterion (e.g., GHG emissions) is equal to the sum of the emissions of all economic sectors, each equal to the number of sectorial workers multiplied by the individual contribution to GHG emissions, defined in the next subsection.

### Data collection and goals’ definition

#### The GDP criterion $F_1$

For a given sector *j* in each country, the sector’s specific economic output per capita $A_{1j}$ is expressed in thousands of euro per capita. In particular, $A_{1j}$ is equal to the ratio of the economic output for the selected sector $GDP_j$ (expressed in millions of euro in 2015, Eurostat 2010, 2014a, b, c, d, e) by the sector’s number of employees $X_j^0$ (expressed in thousands) in the starting year of analysis (OECD Statistics 2015; Eurostat 2010):

3 $$\begin{aligned} A_{1j}=\frac{(GDP)_j}{X_j^0} \end{aligned}$$

The corresponding goal $G_1$ respects the following constraint expressed by Europe 2020 objectives framework: “the economic output must be at least the same”. Then, the economic output objective $G_1$ is simply the predicted sum of the economic output of all sectors for a given country, as estimated by the International Monetary Fund (IMF) and the OECD, over the period 2015–2020. More recent data and forecasts on GDP in European Union countries furnished by the IMF and the OECD are also taken into account for the period 2015–2020. This allows us to provide a calibrated benchmark to compare the achievement of Europe 2020 policy goals, with a reasonably adequate modelling of national economic sectors of EU member countries.

#### The GHG criterion $F_2$

For a given sector *j* in each country, the GHG emission per capita $A_{2j}$ is expressed in tonnes of $\text {CO}_2$ equivalent per capita. In particular $A_{2j}$ is obtained by taking the ratio of the GHG emissions for the selected sector $(\text {CO}_2)_j$ in 2015 (expressed in thousands of tonnes), by the number of employees $X_j^0$ (expressed in thousands) in the same year (OECD Statistics 2015; Eurostat 2010, 2014a, b, c, d, e):

4 $$\begin{aligned} A_{2j}=\frac{(\text {CO}_2)_j}{X_j^0} \end{aligned}$$

The environmental policy target $G_2$ have been computed by applying Europe 2020 objectives, which require a 20% reduction of GHG emissions with respect to 1990 GHG emissions level.

#### The energy criterion $F_3$

For a given sector *j* in each country, the energy consumption per capita $A_{3j}$ is expressed in tonnes of oil equivalent per capita. It has been obtained by taking the ratio of the energy consumption for the selected sector $EC_j$ (expressed in thousands of tonnes of oil equivalent in 2015, International Energy Agency 2014), by the number of employees $X_j^0$ (expressed in thousands) in the same year (OECD Statistics 2015; Eurostat 2010, 2014a, b, c, d, e):

5 $$\begin{aligned} A_{3j}=\frac{(EC)_j}{X_j^0} \end{aligned}$$

In order to compute energy consumption goal $G_3$, we applied proceeded by applying the Europe 2020 objectives, i.e. a reduction of energy consumption by 16.66% with respect to the 1990 level.

#### The employment constraint $F_4$

The model aims to keep employment at least the same in each sector, based on the proportions observed in the year of data collecting (OECD Statistics 2015; Eurostat 2010). Thus, employment targets in each sector defining the set of possible allocations $\Omega$ are obtained my multiplying the current number of workers in each country by the most recent population growth rates.

#### Quantifying external migrant flow in working age

We consider the internal allocation of internal surplus workers, but also the integration of incoming extra EU workers. Such movement is derived from international migrations, i.e., with an extra European origin and European destination. The number of persons denoted “migrants” by age is extracted from the 2017 revision of Trends in International Migrant Stock compiled by the United Nations’ Population Division. More specifically, empirical calibration refers to the data set “International migration flows to and from selected countries” (2015 data according to the 2017 revision). The migrants’ working capacity is assumed for the age range 20–64 following United Nations Department of Economic and Social Affairs classification, yielding the following Table 3.

**Table 3:** External migrant flow in working age

Age class	All	Percentage of working age	20–64
Size	3,600,000	74.3%	2,674,800

### Optimization steps

Considering the EU2020 objectives satisfaction, our two-stage optimization process aims at identifying the differences arising in switching from a pure national perspective (closed plan) to an European perspective (open plan), when dealing with optimal workers allocation. In the closed plan, countries only allocate their own-possibly growing-population of workers among their economic sectors. This constraint is then relaxed in the open plan, where countries in need or in excess of workforce may borrow or lend workers abroad, following the idea that a balance between supply and demand of workers may exist at the European Union scale. We compute and compare the optimal allocations of workers in each case, and outline the difference in the resulting objective function.

In the closed plan, we compute national optimal allocations in the perspective of EU2020 objectives for each county. The decision variable -workers to be allocated- is here restricted to the feasible set containing all the country workers, i.e. those present at the first year of analysis and those from natural population growth. We here consider OECD forecasts and apply the population growth rate to the population of workers, assuming that population growth equally targets the population of workers and the population of non-workers. We impose the constraint that during the optimization process the employment in each sector has to be maintained. As a consequence, the decision of each country depends of the growth rate of its population. Thus, a negative population growth rate impacts drastically on the decision possibilities of the country, that is no longer able to maintain the previous levels of employment across the economic sectors.

In the open plan, we consider the case of cooperation between European Union countries. We compute the ideal national strategies when the social criterion is relaxed, i.e. we let the country borrow or lend some workers to improve its joint satisfaction of Europe 2020 objectives. The resulting deviations from the actual population of the country are included in the problem of minimizing deviations. Indeed, in some cases a country may benefit from the arrival of additional workers to improve its achievement of the targets, while in others an excess of workers may be detrimental to EU2020 goals. So that, we are able to find the ideal plan for the country. Then, its implementation will depend on both the balance between supply and demand of workers at the European Union scale, and the allocation rule of the workers in excess.

The first stage of the model determines the level of objectives achievement obtained both in the closed and the open plan, for each country. The second stage compares the performance of each country in the two plans and determines both the supply of and demand for workers, and the allocation of workers among countries in need. The feasible national strategies under international cooperation are thus obtained, and the resulting satisfaction of Europe 2020 objectives computed.

### First stage: allocation of additional workforce growth

Having described all the ingredients of our framework, we build the first stage standard GP models for each country, considering both the open and closed plan. In these models, we look for the smallest deviation values that allow our four macroeconomic linear criteria to be the closest to the targeted output ($G_i$ in the following constrained optimization (6a)–(6e), (7a)–(7d) and (8a)–(8e)). In addition to Europe 2020 objectives, we constrain the number of employees in each sector, $X_j$, to be at least equal to the level ${\Omega _j^0}$ of the starting year of the analysis. In the closed plan, we add the additional constraint that the sum of allocated workers must be strictly equal to the population target.^1^ These social constraints aim at imposing the goal of preserving employment as much as possible in each economic sector. We proceed then, in the closed plan, considering the following model with 7 economic sectors and 3 criteria (economic, environmental, energy), including the strict social one formulated in terms of the strong equality constraint $\sum _{j = 1}^7{X_j} = \sum _{j = 1}^7{\Omega _j^{2020}}=\Omega ^{2020}$, where $\Omega ^{2020}$ represents the estimated population of the country in the year 2020 (Eq. (6d)). The set $\Omega _j$ defines feasible allocations of workers in sector *j*. Now the minimization of the deviations reads as:

6a $$\begin{aligned} \text {maximize}\quad&\sum _{i=1}^{3}{\delta _i^+ + \delta _i^-} \end{aligned}$$

6b $$\begin{aligned} \text {subject to}\quad&\sum _{j=1}^{7}{A_{ij}X_j+\delta _i^-+\delta _i^+=G_i,\quad \text {for}\; \ i=1,\ldots , 3}, \end{aligned}$$

6c $$\begin{aligned}&X_j \ge \Omega _j, \quad \text {for}\; j = 1, \ldots , 7, \end{aligned}$$

6d $$\begin{aligned}&\sum _{j = 1}^7{X_j} = \sum _{j = 1}^7{\Omega _j^{2020}} \end{aligned}$$

6e $$\begin{aligned}&\delta _i^{\pm } \ge 0,\quad \text {for}\; i=1,\ldots ,\ 3. \end{aligned}$$

where $X_j$ are the input variables representing the decision variables in category *j*, the coefficient $A_{ij}$ states the contribution of the *j*th variable to the achievement of the *i*th criterion, and $\delta _i^-$ and $\delta _i^+$ are the positive and negative deviations with respect to the targeted goal levels, for $i = 1, 2, ..., p$ respectively.

In the open or cooperative plan the minimization of the deviations yields the constrained optimization (7a)–(7d), that includes a relaxed hypothesis with respect to the system (6a)–(6e) for the social goal in the objective function, and an inequality feasibility constraint that allows a more flexible allocation of the workforce. The deviations in the number of workers with respect to the actual population are taken into consideration as a fourth criteria to be optimized among the others. This produces the ideal scenario for each country, as if each country was able to benefit from an infinite supply or demand of workers.

7a $$\begin{aligned} \text {minimize}\quad&\sum _{i=1}^{4}{\delta _i^+ + \delta _i^-} \end{aligned}$$

7b $$\begin{aligned} \text {subject to}\quad&\sum _{j=1}^{7}{A_{ij}X_j+\delta _i^-+\delta _i^+=G_i\quad \text {for}\; i=1,\ldots , 4}, \end{aligned}$$

7c $$\begin{aligned}&X_j \ge \Omega _j, \;\text {for}\; j = 1, \ldots , 7 \end{aligned}$$

7d $$\begin{aligned}&\delta _i^-,\delta _i^+\ge 0 \quad \text {for}\; i=1,\ldots , 4. \end{aligned}$$

We implement these models on each EU country of the European Union targeted by Europe 2020 objectives program. We define as “European Union (EU) members” all countries that are members at the date of January 1st, 2018. We then include in the analysis: Austria, Belgium, Bulgaria, Croatia, Cyprus, Czech Republic, Denmark, Estonia, Finland, France, Germany, Greece, Hungary, Ireland, Italy, Latvia, Lithuania, Luxembourg, Malta, Netherlands, Poland, Portugal, Romania, Slovak Republic, Slovenia, Spain, Sweden and the United Kingdom.^2^

### Second stage: multi-country allocation of the workers in surplus

After computing both the closed and the open allocations for each country, we introduce the possibility of cooperation among EU states. Some countries may be able to reach a better satisfaction of EU2020 objectives through widening their workforce. However, this leads to an increase in total population which may reduce wealth per capita. Then, we assume that each country is willing to cooperate and include new workers as long as the GDP per capita is greater or equal to the per capita wealth reached in the closed case. In other words, cooperation (open plan) must not make a country worse-off compared to the non-cooperating strategy (closed plan). This condition is reasonable in a game-theoretical context as it accounts for the economic wealth of countries, while allowing supranational cooperation.

If there is interest for cooperation, i.e., if there is a possibility for the countries to improve the objective function value while not harming per capita wealth, then the demand for new workers can be computed as a value between 0 (the closed plan) and $\delta ^{+}$ (the positive deviations from the social goal in the open plan). It is intuitive that if per capita wealth and objective function value are higher in the open rather than in the close case, then the demand for workers will be a corner solution at $\delta ^{+}$. On the other way round, if a country is worse-off or indifferent in adopting the open plan or the closed plan, then the demand for workers will be a corner solution at 0. The intermediate case arises when switching to plan open improves the satisfaction of EU 2020 objectives, but lessens per capita wealth. When it is not possible to improve the EU2020 targets achievements without letting per capita wealth fall under the closed threshold, the Pareto efficient demand for workers will be an interior solution between 0 and $\delta ^{+}$. In this second stage we determine this interior solution: for the countries in which the workers demand is not intuitive, we implement the following revised open GP model, adding the constraint that per capita wealth has to remain constant with respect to the value obtained under the closed plan allocation $\pi$. The set $\Omega _j$ defines feasible allocations of workers.

8a $$\begin{aligned} \text {minimize}\quad&\sum _{i=1}^{4}{\delta _i^+ + \delta _i^-} \end{aligned}$$

8b $$\begin{aligned} \text {subject to}\quad&\sum _{j=1}^{7}{A_{ij}X_j+\delta _i^-+\delta _i^+=G_i,\quad \text {for}\; \ i=1,\ldots , 4}, \end{aligned}$$

8c $$\begin{aligned}&X_j \ge \Omega _j,\; \text {for}\; j = 1, \ldots , 7, \end{aligned}$$

8d $$\begin{aligned}&\frac{\sum _{j=1}^{7}{A_{1j}X_j}}{\sum _{j=1}^{7}{X_j}} \ge \pi , \end{aligned}$$

8e $$\begin{aligned}&\delta _i^-,\delta _i^+\ge 0,\quad \text {for}\; i=1,\ldots , 4. \end{aligned}$$

Once we obtain the demand adjusted by the national preferences, we want to maximize the satisfaction of EU2020 goals obtained from the allocation of workers in the European Union countries from the collective perspective allowed by cooperation. In this optimization process, whose details are described in the following constrained optimization (9a)–(9d), the objective function is equal to the sum for all the countries of the product of the number of surplus workers allocated in a country *l*, noted $Y_l$, by a necessity ratio $\psi _l$ specific to the country *l*. As described in Eq. (9c), the necessity factor is obtained as the ratio between the deviation from the employment objective (i.e., the demand for workers $\delta ^{+}$ computed in the constrained optimization (8a)–(8e) and the employment objective $G_4$. The decision maker is then interested in allocating workers in countries where the demand of workers is higher compared to their current population of workers. Thus, for each country *l* in need for additional workforce and in which conditions for cooperation are validated, we have:

9a $$\begin{aligned} \text {maximize}\quad&\sum _{l=1}^{28}{\psi _lY_l}, \end{aligned}$$

9b $$\begin{aligned} \text {subject to}\quad&\sum _{l=1}^{28}{Y_l} = Z, \end{aligned}$$

9c $$\begin{aligned}&\psi _l = \frac{\left( \delta _{4,l}^+ - Y_l \right) - G_{4,l}}{G_{4,l}}, \;\text {for}\; l = 1, \ldots , 28, \end{aligned}$$

9d $$\begin{aligned}&Y_l \le \delta _{4,l}^+, \;\text {for}\; l = 1, \ldots , 28, \end{aligned}$$

where $Y_l$ is the decision variable and corresponds to the number of available workers allocated in country *l*. *Z* is the total number of available workers coming from countries which do not need additional workforce to improve their efficiency in terms of EU2020 objectives or their wealth per capita. Countries in excess of workers, instead, prefer to move these workers to countries in need of additional workforce. These lasts, are the countries with negative deviations from the social criterion $\delta _4^-$, as listed in Table 4. Moreover, our allocation plan to improve sustainability and per capita wealth in the population of workers benefits from adding workers or migrants from outside the European Union, as the demand for workers emerging from the optimal allocation largely exceeds the supply provided by other EU countries. We consider in this work only flows of workers toward, and within the EU.

The constraint $\psi _j = \frac{\left( \delta _{j,42} - Y_j \right) - G_{j,4}}{G_{j,4}}$ introduces dynamic mechanisms in this allocation algorithm. Indeed, the necessity value $\psi _j$ is not static as it depends on its own fulfillment as workers get allocated. Without this adding, the country with the highest necessity value would capture as much workers as possible, even if their marginal utility in addressing the necessity criterion would be decreasing. The inclusion of this dynamic system allows the allocation to take marginal gains in necessity fulfillment into account and results in a more balanced allocation scheme.

## Results

The results for Malta and Cyprus were not included in the representations, as the data analysis procedure for these countries was not complete, due to missing and/or confidential data. In the following Tables, NF stands for not feasible. It means that it was not possible to find an optimal solution (see Sect. 4 for detailed explanations). Per capita GDP is expressed in thousands of euro. Results are marked with an index $^*$ when they are are related to countries for which the trade-off between sustainability performance and per capita wealth needed further investigation done by the means of our adjusted allocation scenario.

**Table 4:** First stage GP model: objective function and per capita wealth in the closed and open plans at horizon 2020

Country	Closed plan		Open plan	
	Obj. function	Per capita GDP	Obj. function	Per capita GDP
Austria	91,913,570	50.54	33,384,420	65.20
Belgium	10,124,420	73.08	10,124,420	73.08
Bulgaria	NF	16.63	851,199.4	16.27
Croatia	NF	27.97	8,052,256	24.08
Czech Rep.	123,157,200	25.06	92,216,490	24.86
Denmark	18,660,730	76.48	5,227,073	78.68
Estonia	NF	24.09	12,967,860	23.91
Finland	42,294,680	61.80	29,816,480	64.00
France	164,756,000	58.58	43,359,290	61.08
Germany	NF	54.40	48,809,080	57.44
Greece	NF	28.50	15,861,620	29.39
Hungary	NF	18.91	1,454,844	18.19
Ireland	35,189,560	89.84	14,377,600	116.89
Italy	NF	48.12	31,249,090	9.83
Latvia	NF	19.79	307,611	19.67
Lithuania	NF	22.75	545,781.6	20.22
Luxembourg	1,939,922	106.62	1,837,285	116.111
Netherlands	10,856,590	52.70	10,856,590	52.70
Poland	NF	39.26	16,125,550	42.90
Portugal	NF	27.46	18,270,250	28.75
Romania	NF	33.98	1,312,043	34.09
Slovak Rep.	540,161,700	18.14	46,349,430	9.41
Slovenia	26,827,650	60.36	25,917,200	60.59
Spain	NF	43.14	124,117,000	44.25
Sweden	6,347,251	73.26	2,095,613	70.90
UK	69,396,490	58.12	18,903,510	59.30

The first stage and second stage optimization process was implemented using the LINGO software, and we present in the following tables our main results and steps of analysis. In the following tables we present the steps of analysis and our main results. In Table 4, we expose the results from the first stage of the GP model. We compare the objective function value and per capita GDP in the population of workers in the closed and open plans.

**Table 5:** First stage GP model: workers supply and demand in the open plan

Country	$\delta _{4}^{-}$	$\delta _{4}^{+}$
Austria		461,114
Belgium		
Bulgaria		851,199
Croatia		449,797
Czech Rep.		66,183
Denmark		96,645
Estonia		64,457
Finland		120,772
France	3,138,957	
Germany		7,292,552
Greece		545,454
Hungary		1,446,852
Ireland	724,718	
Italy		3,965,897
Latvia		307,611
Lithuania		545,781
Luxembourg	38,031	
Netherlands		
Poland		852,255
Portugal		553,387
Romania		1,312,043
Slovak Rep.		4,898,122
Slovenia	18,249	
Spain		342,883
Sweden		171,691
UK	1,283,498	
Sums	5,203,453	24,344,695

Table 5 presents more in detail the respective deviations $\delta _{4}^-$ and $\delta _{4}^+$ for the social criterion, that correspond to the excess and the need of workers in the open (unconstrained) scenario. The importance of our second optimization stage arises from the disequilibrium pattern in the allocation of workforce. Thus, we can introduce the possibility of intra-Europe transfers of workers, to increase collective Pareto efficiency of EU countries, without harming previous national optimal allocations. We then do not assume total submission of the countries to a centralized coordinating entity, or perfect collaboration. Assuming instead perfect mobility of workers in European Union makes sense considering the importance of freedom of movement, capitals and goods in the common free market. In the context of high and increasing migrations towards European countries, and given that the single allocation of European workers in excess does not allow a completer satisfaction of the workforce need, it seems interesting to also consider the possible integration of non-European incoming workers. In the adjusted open scenario, we introduce the constraint of keeping per capita GDP at least equal to the value obtained in the closed plan, for the reasons exposed in the previous section.

**Table 6:** Second stage optimization process: adjusted demand and allocation

Country	$\delta _{4}^-$	$\delta _{4}^+$
Austria		461,114
Belgium		
Bulgaria		803,818$^{*}$
Croatia		91,619$^{*}$
Czech Rep.		66,183 $^{*}$
Denmark		96,645
Estonia		59,964$^{*}$
Finland		120,772
France	3,138,957	
Germany		7,292,552
Greece		545,454
Hungary		1,263,730$^{*}$
Ireland	724,718	
Italy		3,965,897$^{*}$
Latvia		301,466$^{*}$
Lithuania		478,579$^{*}$
Luxembourg	38,031	
Netherlands		
Poland		852,255
Portugal		553,387
Romania		1,312,043
Slovak Rep.		4,898,122$^{*}$
Slovenia	18,249	
Spain		342,883
Sweden		171,691
UK	1,283,498	
Sums	5,203,454	23,678,174

Deviation values that are different from the first stage of the optimization are denoted with *

**Table 7:** Comparison of objective function values for open, closed and adjusted open plans

Country	Closed plan	Open plan	Adjusted open plan
Austria	91,913,570	33,384,420	
Belgium	10,124,420	10,124,420	
Bulgaria	NF	851,199.4	1,638,733$^{*}$
Croatia	NF	8,052,256	9,376,977$^{*}$
Czech Rep.	123,157,200	92,216,490	92,216,490$^{*}$
Denmark	18,660,730	5,227,073	
Estonia	NF	12,967,860	13,086,960$^{*}$
Finland	42,294,680	29,816,480	
France	164,756,000	43,359,290	
Germany	NF	48,809,080	
Greece	NF	15,861,620	
Hungary	NF	1,454,844	1,463,383$^{*}$
Ireland	35,189,560	14,377,600	
Italy	NF	31,249,090	31,249,090$^{*}$
Latvia	NF	307,611	485,118$^{*}$
Lithuania	NF	545,781.6	4,844,028$^{*}$
Luxembourg	1,939,922	1,837,285	
Netherlands	10,856,590	10,856,590	
Poland	NF	16,125,550	
Portugal	NF	18,270,250	
Romania	NF	1,312,043	
Slovak Rep.	540,161,700	46,349,430	352,472,400
Slovenia	26,827,650	25,917,200	
Spain	NF	124,117,000	
Sweden	6,347,251	2,095,613	6,347,306
UK	69,396,490	18,903,510	

Deviation values that are different from the first stage of the optimization are denoted with *

Then, in the second stage, we are allocating 5,203,454 internal and 2,674,800 external surplus workers in the set of 19 European Union countries which are in need of additional workforce. This constrained environment yields new values of demand for workers in countries for which the determination of needs is an interior point, presented in Table 6. Once the adjusted allocation plans are determined, we describe their benefits in objective function values, i.e., Table 7 and per capita GDP levels, i.e., Table 8.

**Table 8:** Comparison of per capita GDP values for open, closed and adjusted open plan

Country	Closed plan	Open plan	Adjusted open plan
Austria	50.54	65.20	
Belgium	73.08	73.08	
Bulgaria	16.63	16.27	16.63$^{*}$
Croatia	27.97	24.08	27.97$^{*}$
Czech Rep.	25.06	24.86	25.06$^{*}$
Denmark	76.48	78.68	
Estonia	24.09	23.91	24.09$^{*}$
Finland	61.80	64.00	
France	58.58	61.08	
Germany	54.40	57.44	
Greece	28.50	29.39	
Hungary	18.91	18.19	18.91$^{*}$
Ireland	89.84	116.89	
Italy	48.12	9.83	51.16$^{*}$
Latvia	19.79	19.67	19.79$^{*}$
Lithuania	22.75	20.22	22.75$^{*}$
Luxembourg	106.62	116.111	
Netherlands	52.70	52.70	
Poland	39.26	42.90	
Portugal	27.46	28.75	
Romania	33.98	34.09	
Slovak Rep.	18.14	9.41	18.14$^{*}$
Slovenia	60.36	60.59	
Spain	43.14	44.25	
Sweden	73.26	70.90	73.26$^{*}$
UK	58.12	59.30	

Deviation values that are different from the first stage of the optimization are denoted with *

Finally, Table 9 presents the number of workers allocated per country if we were only allocating internal workforce from our dynamic optimization process. Allowing external workforce in the optimization process simply result in the complete fulfillment of the demand for workers, given that the workers supply in this case exceeds the total number of workers required at optimum by European Union countries and is already captured by the results of the open plan.

**Table 9:** Second stage optimization process: adjusted demand and allocation

Country	$\delta _{4}$	$Y_j$
Austria	461,114	299,165
Belgium	0	0
Bulgaria	803,818$^{*}$	0
Croatia	91,619$^{*}$	0
Czech Rep.	66,183$^{*}$	0
Denmark	96,645	0
Estonia	59,964$^{*}$	0
Finland	120,772	0
France	$-$ 3,138,957	$-$ 3,138,957
Germany	7,292,552	941,832
Greece	545,454	28,096
Hungary	1,263,730$^{*}$	379,359
Ireland	$-$ 724,718	$-$ 724,718
Italy	3,965,897$^{*}$	554,960
Latvia	301,466$^{*}$	96,897
Lithuania	478,579$^{*}$	158,842
Luxembourg	$-$ 38,031	$-$ 38,031
Netherlands	0	0
Poland	852,255	0
Portugal	553,387	0
Romania	1,312,043	449,209
Slovak Rep.	4,898,122$^{*}$	2,295,094
Slovenia	$-$ 18,249	$-$ 18,249
Spain	342,883	0
$$\hbox {Sweden}^{**}$$	$$-$$ 1	0
UK	$-$ 1,283,498	$-$ 1,283,498
Sums	18,474,720	0

Deviation values that are different from the first stage of the optimization are denoted with *

## Discussion

Table 4 shows the values of the objective functions which has to be minimized (as the sum of deviations from the targets) and per capita GDP in 2020, given the optimal allocations for both the open and closed plans. Comparing the objective functions in both scenarios, we can see that the open one guarantees significant gains in term of EU2020 objectives. In particular, 24 out of 26 EU countries show this improvement. Netherlands and Belgium do not improve in the open case, meaning that they already met an optimum in the closed case and did not require cooperation to perform better.

The objective function in the closed case could not be computed for some countries, as solving the GP model generated unfeasible solutions. Specifically, these countries were not able to fulfill the requirement that employment level in each sector had to remain at least constant, concerning the year of analysis. Indeed, as these countries have a negative change in the natural population rate, the objective value $G_4$ was lower than the sum of workers needed to be maintained in economic sectors. That is why non-feasibility occurred. This result highlights the impossibility of reaching this objective in the closed plan and that a cooperative (open) approach would thus be recommended.

Looking at per capita GDP in both plans is insightful. First, from Table 4, we observe that Netherlands and Belgium do not change the optimal per capita GDP, as they reached the optimal in the closed plan. On the one hand, in Austria, Denmark, Finland, France, Germany, Greece, Ireland, Luxembourg, Poland, Portugal, Romania, Slovenia, Spain and United Kingdom (14 countries), the open plan guarantees a higher per capita wealth among workers. This result, combined with the improvement in the objective function, shows that these countries would be better-off in terms of wealth by cooperating. On the other hand, in Bulgaria, Croatia, Czech Republic, Estonia, Hungary, Italy, Latvia, Lithuania, Slovak Republic and Sweden (10 countries), the open plan reduces per capita wealth. Then, the achievement of EU2020 objectives is better in such a plan. Thus, there is a trade-off between cooperation (open plan) and national strategies (closed plan).

In the two-stage optimization approach, we are interested in the behaviour of national job markets. Table 5 presents the negative $\delta _{4}^-$ and positive $\delta _{4}^+$ deviations from the social objective $G_4$ as described in earlier sections. A positive value of $\delta _{4}^-$ means that the optimal allocation of workers for a given country leaves some of them unemployed. In other terms, in the global perspective of multi-criteria decision-making, it is more efficient for these countries not to employ all the available workers concerning Europe 2020 different goals. On the opposite, a country with positive value of $\delta _{4}^+$ needs additional workers to implement the optimal allocation. The results of the first stage GP model show that European countries (except The Netherlands and Belgium) are looking for new workforce. Moreover, a higher supply of workers would increase Europe 2020 achievements in the majority of countries.

The improvement (reduction) in objective function values are described in Table 7. In Bulgaria, Croatia, Estonia, Latvia, Lithuania, Hungary and the Slovak Republic, the objective function value, i.e., the sum of deviations from EU2020 targets that we want to minimize, is higher in the adjusted plan than in the open plan. In Hungary, we observe this result with a minimal difference between the two plans. For the Czech Republic and Italy, the objective function value remains constant in the adjusted case against the pure open one. These results are not surprising, given that the adjusted scenario is more strict compared to the pure open one. Moreover, it puts emphasis on per capita GDP which can counterbalance the objective of minimizing deviations from EU2020 sustainability goals. However, let us notice that for some countries the objective function value is lower in the adjusted open plan than in the closed plan, meaning that it would be more efficient in the perspective of sustainability goals to switch to the adjusted open plan.

As there is no loss in the second criterion (per capita GDP), cooperating would be profitable for the Slovak Republic in our framework. We can apply the same reasoning to countries where the objective function value could not be determined as the problem was not feasible in the closed plan, namely Bulgaria, Croatia, Estonia, Italy, Latvia, and Lithuania. Let us stress that Sweden would get worse performance in achieving EU2020 in the adjusted case rather than in the closed one by a minimal margin.

Table 8 compares per capita GDP in different scenarios. Only Italy shows a clear interest of adopting the adjusted policy plan for this criterion. In the other countries, for which computing the adjusted open plan was relevant, we find no improvement as the adjusted open plan is only able to keep per capita GDP constant. Moreover, this also means that the problem was feasible for all countries given this constraint. So, it is possible to improve EU2020 goals satisfaction, while keeping per capita GDP constant among workers, even if the latter implies a reduced performance in the former.

Therefore, some countries would be willing to adopt the adjusted open plan, as cooperating grants a Pareto improvement by 2020. In particular, for Austria, Denmark, Finland, France, Germany, Greece, Ireland, Luxembourg, Poland, Portugal, Romania, Slovenia, Spain and the United Kingdom, cooperation increases both per capita GDP and sustainability performance. In Italy, the cooperation plan increases per capita wealth and keeps EU2020 objectives achievement constant; in the Slovak Republic, cooperation would imply as well better objectives achievement without harming per capita GDP. The same result from implementing the feasible open plan implies the same consequences in Bulgaria, Croatia, the Czech Republic, Estonia, Hungary, Latvia, Lithuania. Differently, Sweden would not cooperate, as the open adjusted scenario would imply lower per capita GDP and (slightly) worse multi-criteria sustainability. Finally, Belgium and Netherlands do not gain in any criterion by adopting the open plan.

Table 9 shows the demand and supply for workers in the open plan, given the wealth constraint. In particular, the second column displays the net results of the dynamic allocation algorithm introduced in the constrained optimization (9a)–(9d). The fulfillment of the necessity criterion is optimized with the following division: five countries, namely France, Ireland, Luxembourg, Slovenia, United Kingdom which have a surplus of workers, let these persons to migrate. On the contrary, Belgium and the Netherlands do not change anything, as they already reach the Pareto optimal point. Finally, Sweden is not cooperating. Our algorithm allocates most of surplus workers to Slovak Republic, which is consistent with our previous insights, as this country would benefit both in per capita GDP and satisfaction of EU2020 goals. Germany and Italy follow with more than 500,000 workers allocated, which can be explained by the negative natural population growth rate. Then, in smaller amounts, Romania, Hungary, Austria, Lithuania, Latvia and Greece gain some workers from the allocation. Others countries do not receive any additional workers in this model, either since they are not part of the cooperation, or because the marginal fulfillment of the necessity criterion is lower than in other countries.

## Conclusion

Facing the current increase in migration flows towards Europe due to economic, political and international contexts, the transition towards sustainable development and reduction of environmental impact, cornerstone of Europe 2020 policy goals, cannot ignore these migration flows. Moreover, the European Union constitutes a regional organization enforcing free mobility of capital and persons. Thus, we investigate the consequences of intra-Europe (workers flows) and extra-Europe (immigration flows) in the model over the satisfaction of Europe 2020 policy targets. The main contribution of this work is to include migration flows in the design of multi-criteria policies.

We introduce a two-stage multi-criteria decision process. In the first stage, national and cooperative optimal allocations are implemented to reach the highest multi-criteria sustainability performance in the context of EU2020 objectives. We extend our analysis to all European Union countries, to study the possible benefits of cooperation through collective management of workers flows. Indeed, EU2020 targets and maximization of per capita GDP in some countries are satisfied by adding workers, and by reducing them in others. The surplus workers are allocated across countries to address a dynamic necessity criterion and to maximize the marginal gains from the allocation among cooperating countries. Our analysis shows that cooperation is profitable in these terms for almost all European Union members. Apart from few exceptions, EU countries are better off by introducing free movements of workers within the European Union and following supranational allocation strategies in the achievement of Europe2020 sustainability targets.

Our analysis shows that in order to fully implement the ideal open plan, the addition of 18,474,720 workers would be beneficial to the multi-criteria objective performance of EU countries, in line with previous analyses in the Europe 2020 framework (Vié et al. 2018; Liuzzi et al. 2020). This additional inflow of workers would be beneficial both in term of per capita GDP and/or EU2020 objectives satisfaction. This demand would be partially fulfilled by the integration of workforce immigrating from outside the European Union. This represents a yearly flow of 2,674,000 migrants in working age with Europe as main destination (following United Nations Trends in International Migrant Stock and data for the year 2015).

In-flows are empirically counter-balanced by out-flows, i.e., migration of workers from the European Union. Our model data is based on incoming flows. Certainly, taking out-coming flows is necessary for a precise assessment of the optimal allocation of surplus workforce. However, we believe that this simplification impacts marginally the workforce allocation, and does not affect the main findings of this work.

The GP models provide a tractable framework for evaluating policy-making outcomes involving conflicting objectives. This method also relies on simplifying assumptions. Our set-up assumes that workers can freely move across economic activities, regardless of education, skills and preferences. Hence, our results suggest that policy-makers should consider the importance of facilitating movement across sectors, creating a more flexible job market. Nowadays, these suggestions are crucial, especially in light of the economic turmoil after the Covid-19 outbreak.

This analysis highlights the crucial role of workers free movement in implementing supranational allocation plans, that are themselves relevant to tackle the issues arising from the multiplicity of objectives, notably economical and environmental. National performance and policy recommendations are thoroughly presented and discussed. Additionally, the inclusion of foreign workforce in national economic systems outlines the parallel recommendation to ensure adequate workforce integration in national economic sectors, as well as professional training and skills acquisition policies. Targeted sectorial and educational investments are key means of action to efficiently allocating the incoming workforce from population growth as well as migration and worker flows in order to achieve Europe 2020 sustainable development goals. These findings outline the policy relevance of MCDA approaches to economic development challenges, and open to further research on implementation of these sectorial investments.

## Footnotes

1 This is composed of the actual population to which is applied the natural growth rate.

2 Due to missing and/or confidential data in Eurostat database, we do not include Malta and Cyprus in the results presentation.
